# A Brief Photocatalytic Study of ZnO Containing Cerium towards Ibuprofen Degradation

**DOI:** 10.3390/ma14195891

**Published:** 2021-10-08

**Authors:** Alexandro S. Sá, Rodrigo P. Feitosa, Luzia Honório, Ramón Peña-Garcia, Luciano C. Almeida, Juliana S. Dias, Lorena P. Brazuna, Thiago G. Tabuti, Eduardo R. Triboni, Josy A. Osajima, Edson C. da Silva-Filho

**Affiliations:** 1 LIMAV, Interdisciplinary Laboratory for Advanced Materials, Ministro Petronio Portela, Federal University of Píaui, Teresina 64049-550, Piaui, Brazil; alexsousasa@hotmail.com (A.S.S.); rooprado@ufpi.edu.br (R.P.F.); luzia_quimica@yahoo.com.br (L.H.); rraudelp@gmail.com (R.P.-G.); 2 Academic Unit of Santo Agostinho, Federal Rural University of Pernambuco, Recife 52171-900, Pernambuco, Brazil; 3 Chemical Engineering Department, Federal University of Pernambuco, Recife 52171-900, Pernambuco, Brazil; luciano.calmeida@ufpe.br; 4 Laboratory of Nanotechnology and Process Engineering, Chemistry Engineering Department, University of São Paulo, Lorena 12602-810, São Paulo, Brazil; julianasilvaddias@gmail.com (J.S.D.); lorenaamelo1@gmail.com (L.P.B.); thiagogaleote@gmail.com (T.G.T.); tribonier@usp.br (E.R.T.)

**Keywords:** drug, photodegradation, remediation, semiconductor

## Abstract

Ibuprofen (IBU) is one of the most-sold anti-inflammatory drugs in the world, and its residues can reach aquatic systems, causing serious health and environmental problems. Strategies are used to improve the photocatalytic activity of zinc oxide (ZnO), and thosethat involvethe inclusion of metalhave received special attention. The aim of this work was to investigate the influence of the parameters and toxicity of a photoproduct using zinc oxide that contains cerium (ZnO-Ce) for the photodegradation of ibuprofen. The parameters include the influence of the photocatalyst concentration (0.5, 0.5, and 1.5 g L^−1^) as well as the effects of pH (3, 7, and 10), the effect of H_2_O_2_, and radical scavengers. The photocatalyst was characterized by Scanning Electron Microscopy-Energy Dispersive Spectroscopy, Transmission electron microscopy, Raman, X-Ray Diffraction, surface area, and diffuse reflectance. The photocatalytic activity of ibuprofen was evaluated in an aqueous solution under UV light for 120 min. The structural characterization by XRD and SEM elucidated the fact that the nanoparticle ZnO contained cerium. The band gap value was 3.31 eV. The best experimental conditions for the photodegradation of IBU were 60% obtained in an acidic condition using 0.50 g L^−1^ of ZnO-Ce in a solution of 20 ppm of IBU. The presence of hydrogen peroxide favored the photocatalysis process. ZnO-Ce exhibited good IBU degradation activity even after three photocatalytic cycles under UV light. The hole plays akey role in the degradation process of ibuprofen. The toxicity of photolyzed products was monitored against *Artemia salina* (bioindicator) and did not generate toxic metabolites. Therefore, this work provides a strategic design to improve ZnO-Ce photocatalysts for environmental remediation.

## 1. Introduction

Pharmaceutical products are consideredemerging pollutants in numerous aquatic matrices such as waste, surface, and groundwater [1]. They are considered semi-persistent pollutants due to their resistance, consumption, and non-volatile character [2]. The toxicity, high biological activity, and formation of dangerous metabolites can lead to serious and long-term problems for aquatic organisms and water quality [3]. In addition, their ability to be absorbed generates uncertainties about their effects on metabolism and persistence in the body of living beings and ecosystems [4]. The Handbook of Environmental Chemistry emphasizes the danger of these compounds by stating: “every day new potential emerging contaminants are discovered and new disinfection by-products are also generated during treatment, with total ignorance of their potential toxicity or effect on human health” [5].

Antibiotics, analgesics, anti-inflammatory drugs, beta blockers, and others are found in water bodies, which reinforces the need for oxidation technologies [6]. Jiménez-Salcedo et al. [7] investigated compounds that are detected in wastewater at concentration levels up to 52 μg L^−1^. Among non-steroidal anti-inflammatory drugs (NSAIDs), ibuprofen (IBU) is one of the most sold in the world [8] and is used to treat muscle pain, headaches, and fever [9]. Toxicity and IBU concentration in water bodies has been seen as a biological hazard caused by the difficulty of removing it due to the ineffectiveness of water treatment. Carballa et al. [10] indicated that IBU can be removed by conventional biological treatments, reaching up to 70% removal.

Advanced oxidative processes (AOPs), specifically heterogeneous photocatalysis, are an alternative for the degradation and mineralization of several persistent organic pollutants in water bodies [8]. Heterogeneous photocatalysis is a versatile and environmentally friendly technology that uses solar irradiation (UV-vis-IR spectral range) at room temperature and pressure conditions, favoring photoreactions that generate few by-products and allow the recycling of the photocatalysts [11]. Degradation of IBU has been carried out by ozonation processes [12], photo-fenton [13], and photocatalysis [14].

Zinc oxide (ZnO), due to its physicochemical stability, has low cost and toxicity [15]. ZnO has band gap energy of around 3.0–3.2eV, which implies fast excitonic recombination, decreasing its photocatalytic performance [16]. Strategies used to improve the photocatalytic activity of ZnO mainly decrease the excitonic recombination [17,18]. A very efficient method is the introduction of metal in the matrix network, thus forming new electronic levels in the semiconductor that enable charge transfer processes and can reduce recombination (e^−^/h^+^) [19]. Transition metals such as Fe, Ni, Cu, and Ce are used to improve the properties of ZnO [20]. Cerium receives special attention for enabling the formation of oxygen vacancies and acting as an electron-capturing agent [21].

Discharges of toxic pollutants into the aquatic environment are a major threat to living organisms. As toxicity usually refers to the level of a toxic chemical, several conventional physicochemical parameters are used to assess the environmental risk of the pollutant [22]. The incomplete oxidation of pollutants can generate intermediates of a more toxic nature than the original compound [23,24]. The toxicity test is used during different stages of the AOPs treatment and employs different organisms, for example fish, bacteria, algae, plants, and microcrustaceans [25]. *Artemia salina* is a commonly used model to assess the toxicity of various pollutants and is a widely accepted alternative because it is a simple, fast, and economical method, making it attractive in many studies and toxicity analyses [22,26]. The advantage of employing these microcrustaceans is due to their natural tolerance to a varietyof abiotics in the environment [27]. Therefore, the use of *Artemia salina* as a model to assess the aquatic toxicity of pollutants has increased in recent years for the control and generation of toxicity [28].

The innovation ofthis work is the evaluation of the photocatalytic potential of ZnO-Cein the degradation of ibuprofen under ultraviolet light. Operational parameters that interfere in the photocatalytic process were investigated, including the concentration of the photocatalyst, the effect of pH, and the presence of the oxidant H_2_O_2_. To understand the main radical species involved in the photodegradation process, elimination reagents (Ethylenediaminetetraacetic acid, AgNO_3_, and CH_3_OH) were used. Furthermore, the photo-generated effluents based on the monitoring of toxic effects were studied through the use ofa microcrustacean as a test bioindicator, and the recyclability through recovery cycles wasevaluated. Our work provides an important kinetic photocatalytic study, which is necessary for future studies and applications in environmental protection.

## 2. Materials and Methods

### 2.1. Chemicals

Ibuprofen (≥98%, C_13_H_18_O_2_) was obtained from Orn Pharma Nostra (Teresina, Brazil). The reagents used in the synthesis of photocatalysts—gycerol, Zn(NO_3_)_2_, (NH_4_(Ce)_4_NO_3_, Zinc acetate dihydrate (C_4_H_6_O_4_Zn·2H_2_O)—were purchased from Sigma-Aldrich (São Paulo, Brazil) and were used as the precursor for the synthesis of zinc oxide. HCl and NaOH (Sigma Aldrich, São Paulo, Brazil) were used to adjust the pH. Methanol (≥99.6%), silver nitrate (≥99%), and (Ethylenedinitrile)tetraacetic acid (EDTA) (≥98.5%) were purchased from Sigma Aldrich(São Paulo, Brazil). For toxicity assays, the synthetic saline was prepared with magnesium sulfate 98% (Isofar, São Paulo, Brazil), Calcium chloride 99% (Dinâmica), Magnesium chloride 99% (Impex), Sodium bicarbonate 99.4% (Aldrich, São Paulo, Brazil), and Sodium chloride 99% (Dinâmica, São Paulo, Brazil). All reagents were used without prior purification. 

### 2.2. Synthesis of Photocatalyst

The synthesis of ZnO-Ce was previously reported by Dias et al. [29], whereby a 3:1 (mol) solution of glycerol:urea containing Zn(NO_3_)_2_ (0.05 mol) and NH_4_(Ce)_4_NO_3_ (0.01 mol) was prepared in 30 mL under stirring at 70 °C. Afterwards, 5 mL of a solution of sodium hydroxide (0.10 mol) was added slowly, and the white precipitate was filtered. Then, the material named ZnO-Ce was washed and oven dried at 60 °C.

### 2.3. Characterization

The semiconductors were characterized by X-Ray diffraction (XRD) using a Bruker diffractometer (D8 Advance) with Cu-Kα radiation and a scanning rate of 2° min^−1^. Morphology was examined by High-resolution transmission electronic microscopy (HRTEM) images obtained using a JEM-2100-JEOL microscope with a 0.23 nm resolution point and an 80 to 200 kV accelerating voltage. The images were analyzed using Image J software to obtain the average size of the nanoparticles. Scanning electron microscopy (SEM) images were obtained using a field emission electron microscope JEOL JSM-7401F at an acceleration tension of 5.0 kV, an SEI secondary electron detector, a working distance ranging from 3.0 mm, and a resolution of 1.5 nm, with EDS.The analysis of Diffuse Reflectance Spectroscopy (DRS) wasperformed by a Shimadzu spectrophotometer, Model UV-3600 with a diffuse reflectance accessory monitoring in the region of 200 to 800 nm. The optical properties of the synthesized semiconductors were performed on a spectrophotometer UV-Vis spectrometer Shimadzu (UV-2550) and the band gap value was determined based on the standard Kubelka–Munk model [27,30]. The textural properties of the solids were investigated by adsorption–desorption analysis of N_2_ at 77 K using the Quanta chrome Autosorb-iQ Instruments equipment. Thus, before each analysis, approximately 200 mg of the sample was degassed for 4 h at 200 °C. The surface area and pore volume and diameter were calculated using the Brunauer–Emmett–Teller (BET) method based on N_2_ adsorption–desorption. Raman spectra were obtained at 25°C using a spectrophotometer, WITEC brand, Confocal model, using an Ar laser as the excitation source with 50 mW at 533 nm, and spectra were achieved in the region of 200 to 800 cm^−1^.

### 2.4. Photocatalytic Test

The photocatalytic activity of ZnO-Ce was investigated by degradation of the drug ibuprofen in an aqueous solution at 25 ± 1 °C under UV light (125 W Hg without bulb). In a typical experiment, IBU (20 ppm) was tested at photocatalyst concentrations (0.5 gL^−1^; 1.0 gL^−1^; and 1.5 gL^−1^) under magnetic agitation. The potency of the lamp was monitored by a radiometer (HANNA—HI 97500—Luxmeter). The suspension was removed at different times (0, 5, 10, 15, 30, 45, 60, 90, and 120 min). After irradiation, the samples were immediately centrifuged, then absorbance measurements were conducted in a CARY 300 model spectrophotometer. The degradation rate of IBU was determined using Equation (1):

Degradation rate (%) = [(A_0_ − A )/ A_0_] × 100% (1)

A_0_ represents the absorbance in initial time; A is the absorbance of IBU after irradiation. The kinetic study was conducted using the Langmuir–Hinshelwood model, and the rate constant, *k*, was determined under pseudo-first order [31].

#### OperationalParameters in Photocatalytic Test

To monitor photocatalytic activity, we investigated the effect of pH and H_2_O_2_, and the scavengers were investigatedto understand the role of radicals in the photocatalytic process. The pH was around 3.7 to 10, and methyl alcohol (3.4 × 10^−3^ mg/L), silver nitrate (5.0 × 10^−4^ mol/L), and EDTA (2.4 × 10^−6^ mol/L) were used as scavengers of hydroxyl radicals, electrons, and holes, respectively [27,32]. The amounts and concentrations of scavengers used were established according to previous studies [27,33]. The effect of hydrogen peroxide on the photodegradation of IBU in the 20 mgL^−1^ solution was studied by adding 0.5 mmol/L of H_2_O_2_ using 0.5 gL^−1^ of the catalyst. The tests were carried out under the same conditions as the photocatalytic tests, only adding the scavengers or H_2_O_2_ at the described concentrations.

### 2.5. Artemia salina Bioassays

The toxicity of the IBU solution irradiated in bioassays with *Artemia salina* wasdetermined [34] with adaptations according to the procedure described by Araújo et al. [27,33]. The microcrustaceans were obtained after 48 h of cultivation in a synthetic saline solution under continuous illumination and oxygenation. Synthetic saline was obtained by adding salts to ultrapure water as described in Materials. The nauplius wasadded to asolution containing irradiated IBU and synthetic saline solution (1:1 *v/v*), and the mortality of microcrustaceans was evaluated after 24 h and 48 h.

## 3. Results

### 3.1. Characterization

**Figure 1:**
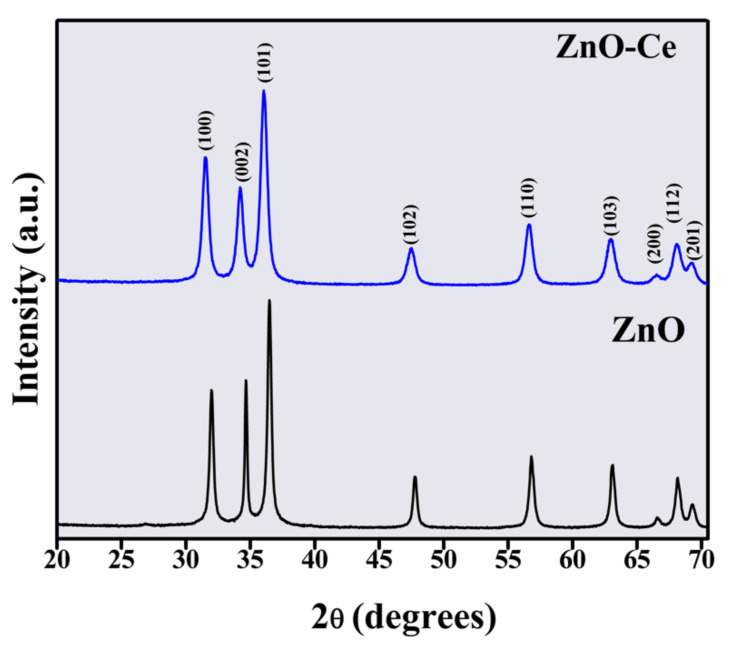
XRD patterns for ZnO and ZnO-Ce.

To verify the phase formation, X-ray diffraction measurements were performed on the samples and the results are shown in Figure 1. The diffraction peaks around 31.4°, 34.2°, 36.4°, 47.7°, 56.7°, 63.0°, 66.6°, 68.0°, and 69.2° correspond to the planes (100), (002), (101), (102), (110), (103), (200), (112), and (201), respectively, and confirm the hexagonal wurtzite structure of ZnO(JCPDS No. 79–2205) [35,36]. No secondary or undesirable phases were observed for the ZnO-Ce sample, suggesting its incorporation in the ZnO hexagonal structure. For both samples, the lattice constants *a* and *c* were calculated using Equation (2) [37]:

(2) $${\left(\frac{1}{d_{hkl}}\right)}^{2}=\frac{4}{3}\left(\frac{h^{2}+k^{2}+hk}{a^{2}}\right)+\frac{l^{2}}{c^{2}}$$

The dhkl values for each (hkl) family can be calculated by the Bragg formula, λ = 2d sin(θ), where θ is the Bragg angle and λ is the incident X-ray wavelength. The lattice parameters a and c for the ZnO sample are 3.2274 Å and 5.1708 Å, respectively, while for the ZnO-Ce sample, they are 3.2843 Å and 5.2420 Å, respectively. Comparing the lattice constant values of the ZnO sample with the ZnO-Ce sample, there was a significant increase, which may be associated with the differences between the ionic radii of Zn (0.74 Å) and Ce (0.97 Å) [38]. On the other hand, the average crystallite sizes (*D*) and the lattice strain (ε) of samples were estimated using the Williamson–Hall equation [37]. For ZnO and ZnO-Ce samples, the *D* and ε values are 22 nm and 12 nm and 0.21 × 10^−3^% and 0.28 × 10^−3^%, respectively. Evidently, the presence of Ce in the ZnO hexagonal structure causes a decrease in the average crystallite size, which may be related to the nucleation rate modification during the ZnO crystallization. If the retarding force is greater than the driving force for growth, a decrease in the average crystallite size is expected [39].

**Figure 2:**
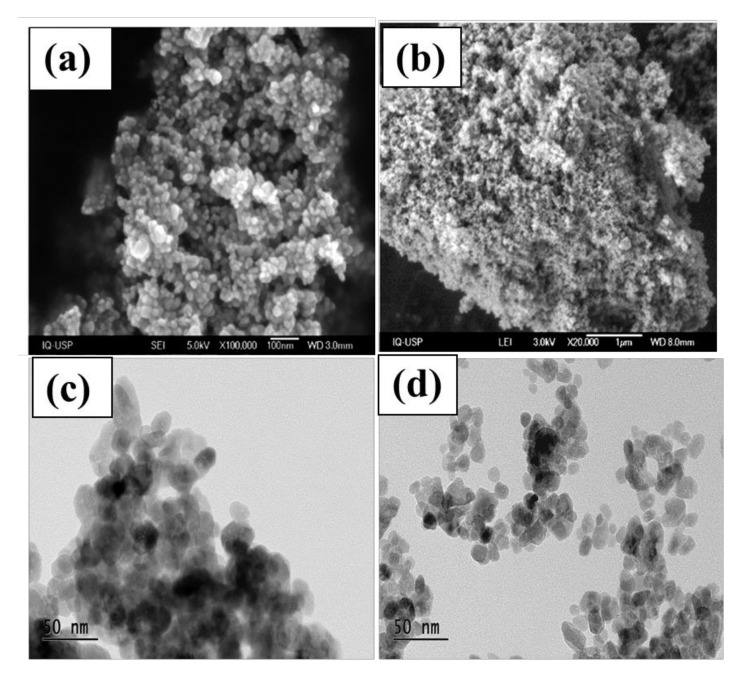
Morphology illustrated by SEM images ZnO (**a**) and ZnO-Ce; (**b**) TEM images of ZnO (**c**,**d**).

SEM images of ZnO and ZnO-Ce are presented in Figure 2. ZnO displays spherical structures with uniform size and distribution (Figure 2a), while for ZnO-Ce, nanoparticles are smaller and more agglomerated (Figure 2b) due to the size of the nanoparticles. The TEM images of ZnO are shown in Figure 2c,d. The presence of cerium in the synthesis of ZnO nanoparticles changes the texture and aggregation of the nanoparticles [31], mainly due to the modification of surface charge densities and the alteration of the crystalline planes of ZnO. The EDS and elemental mappings of ZnO-Ce are displayed in the supplementary material, showing the distribution of cerium in ZnO (Figure S1).

**Figure 3:**
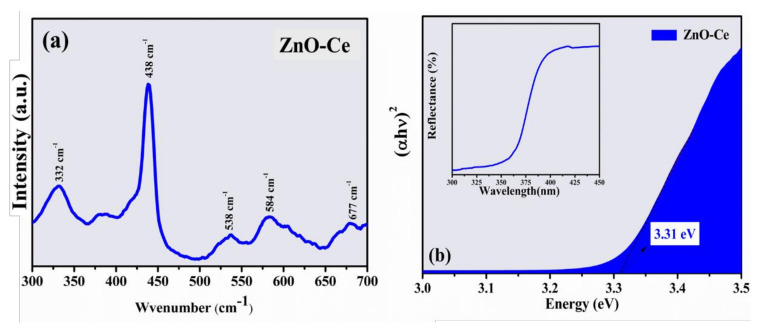
(**a**) Raman spectrum of ZnO-Ce. (**b**) Tauc’s plot to determine the band gap energy of ZnO-Ce. The inset displayed the diffuse reflectance spectra.

The Raman spectrum of ZnO-Ce is shown in Figure 3a. ZnO-Ce exhibited an intense absorption band centered at 438 cm^−1^ (E2H), which is attributed to the main vibration mode of the ZnO phase with a hexagonal wurtzite structure [40], indicating good crystallization and a defined peak. Additional bands are found at 332 cm^−1^, 382 cm^−1^, 538 cm^−1^, 584 cm^−1^, and 677 cm^−1^, also related to active polar modes in Raman attributed to polar modes in ZnO [40]. The peak at 332 cm^−1^ is attributed to second-order Raman scattering, coming from the phonon limit zone 3E2H-E2L, while the peak at 382 cm^−1^can be attributed to the A1T mode [40]. There is a weak band around 584 cm^−1^ (phonon E1L), which may be related to the formation of different defects, such as oxygen vacancies and interstitial Zn [41,42]. Usually, low-intensity bands are identified in materials containing metal due to the network distortion promoted by the presence of metal. 

Some properties directly influence photocatalytic processes, such as the band gap value, because it determines the excitation region of the material. The analysis of absorption spectroscopy in the ultraviolet and visible region (Figure 3b) allows the calculation of the band gap, which is a very important physical characteristic of materials as it affects their electrical behavior [43]. The band gap value of ZnO is one of the most important oxides due to its unique physical characteristics of wide and direct gap (~3.44 eV) with a large exciton binding energy [44]. The Eg value obtained for ZnO-Ce was estimated at 3.31 eV with no significant changes compared to ZnO. ZnO-Ce has a band around 377 nm in the UV-Vis region, with this being the region with the highest absorption energy referring to the transitions constituted mainly by the 2p orbital of oxygen (O) and the 4f orbital of cerium (Ce), resulting in a difference between the energies of each orbital and band gap [45]. 

Another factor is that ZnO-Ce has a surface area of 24.807 m^2^g^−1^, which is higher than ZnO, according to Zhang et al. [46] and other works [47,48,49]. ZnO-Ce has a pore volume of 0.103 cm^3^g^−1^ and has anaverage pore size of 9.369 nm; thus, ZnO-Ce exhibits more active sites, providing greater amounts of reactions, consequently improving photocatalytic performance. Table 1 shows the specific surface of ZnO-Ce and other materials.

### 3.2. Photocatalytic Degradation of IBU

**Figure 4:**
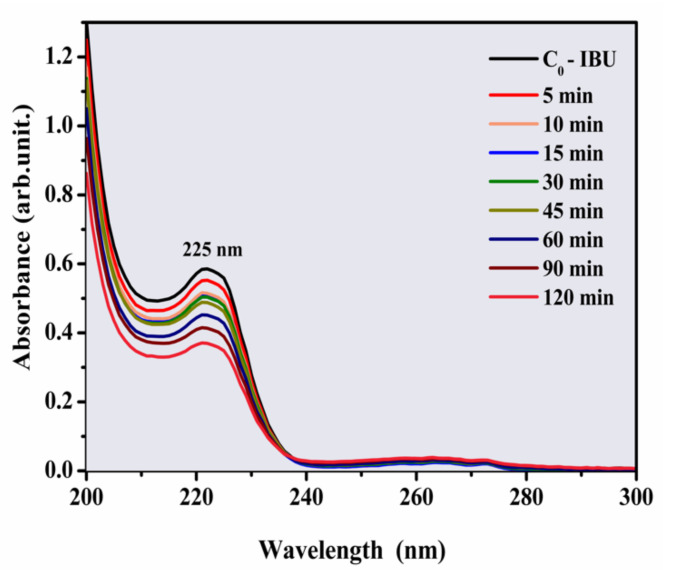
Photolysis of IBU (20 ppm) at different irradiation times under UV light.

Preliminarily, the photolysis of IBU was investigated (Figure 4). The electronic absorption spectra of IBU (20 ppm) showed a degradation rate of 37% after 120 min under UV light. 

**Figure 5:**
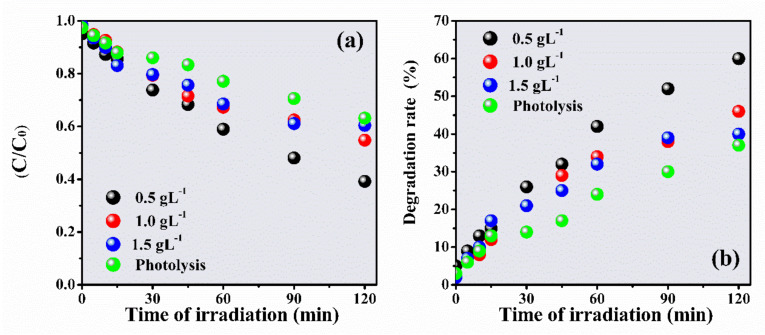
C/C_0_ rate for IBU degradation at different concentrations of ZnO-Ce as a function of radiation time (**a**) and degradation rate (**b**). Experimental conditions: IBU initial concentration: 20 ppm, photocatalyst: 0.50 gL^−1^, UV light: 125 W Hg without bulb.

The degradation of IBU was investigated at different concentrations of ZnO-Ce (Figure 5) because the quantity of the photocatalyst is important for catalytic performance [53]. The IBU efficiencies were 60%, 46%, and 40% at concentrations of 0.5, 1.0, and 1.5 gL^−1^, respectively. IBU degradation was assisted by the Langmuir–Hinshelwood model (a first-order kinetic model) and *k* values for the respective dosages (0.5, 1.0, and 1.5 gL^−1^) obtained at 6.86 × 10^−3^ min^−1^, 4.54 × 10^−3^ min^−1^, and 3.99 × 10^−3^ min^−1^ [54].

The photodegradation of IBU was inversely proportional to the photocatalyst concentration—the lower the ZnO-Ce concentration, the higher the degradation rate. The photocatalyst promoted a higher number of active sites, consequently increasing the concentrations of hydroxyl radicals and superoxides [55]. However, the radicals can recombine and inhibit the photocatalytic process, and a large amount of suspended material can cause the inhibition of light passage, implying a decrease in the photocatalytic efficiency [56]. Similar behavior was reported by Wang et al. [9].

The material can transfer conduction band (CB) electrons from ZnO to cerium due to the larger relative band gap potential of ZnO. This decreases photocatalytic recombination and consequently increases the half-life of the electron species/photo-transferred holes to the photocatalyst surface and contributes to redox reactions. Furthermore, the excessive addition of the photocatalyst has been shown to exceed the synergistic interactions between cerium and ZnO, slowing the process [57].

When ZnO-Ceis irradiated under UV light, the electron excitation favors the formation of photoinduced electrons and holes that generate hydroxyl radicals (HO^•^) and peroxyls (O_2_^•−^) in the photoreactional process. The photoreactional system of ZnO:Ce^4+^ is demonstrated in Equations (3)–(7) [58].

ZnO-Ce+ *h*v→ZnO-Ce(e^−^_CB_ + h^+^_VB_) (3)

h^+^ + H_2_O_ads_→ HO^•^ + H^+^ (4)

h^+^ + OH_ads_→ HO^•^ (5)

e^−^ + O_2_→ O_2_^•−^ (6)

O_2_^•−^ + H^+^→ HO_2_^•^ (7)

**Figure 6:**
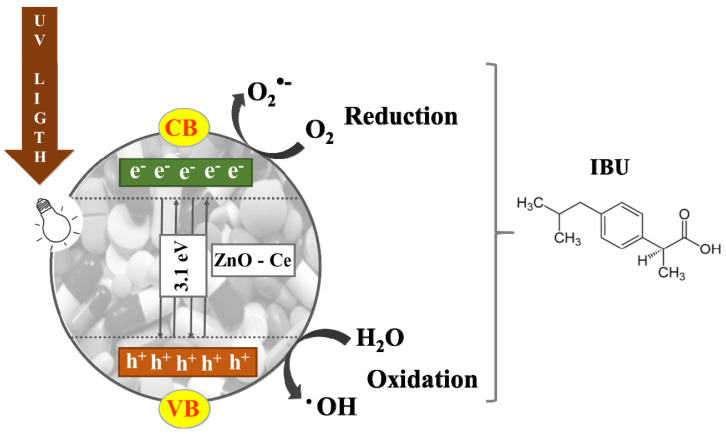
The main photocatalytic reaction using ZnO-Ce in the degradation of IBU.

The main radical reactions generated from the photocatalytic process using ZnO-Ce in the degradation of IBU are presented in Figure 6.

#### 3.2.1. Effect of pH

The pH of heterogeneous photocatalysis is one of the key factors inits operability and yield [55]. Ahigh or low concentration of protons (or hydroxyls) can act on the functional groups of the molecule and the surface of the catalystto be degraded. This can interfere with the adsorption of pollutants, the photogeneration of hydroxyl radicals, and the presence of clusters [59].

**Figure 7:**
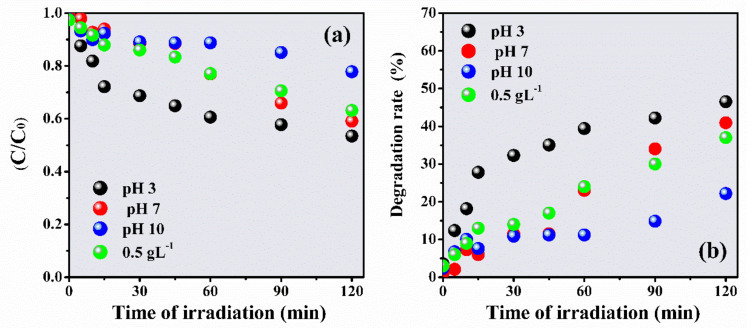
C/C_0_ rate for the degradation of IBU in the presence of ZnO-Ce at different pH as a function of irradiation time (**a**) and degradation rate (**b**). Experimental conditions: IBU initial concentration: 20 ppm, photocatalyst: 0.50 gL^−1^, UV light: 125 W Hg without bulb.

To evaluate the effect of pH on IBU degradation, photoreactions were conducted at pH 3, 7, and 10 (Figure 7). The pH of the initial IBU solution measured was around pH 6.0 (natural solution, without pH adjustment). The degradation rate increased with the increase in the number of protons, following the order pH 10 (22%), pH 7 (41%), and pH 3 (47%); therefore, it increased by about 25% compared to the alkaline medium. The low degradation at pH 10 can be attributed to competition between the surface charges of the photocatalyst, which are slightly altered and may be protonated/deprotonated, consuming the hydroxyl radical and consequently decreasing the degradation rate [56,60]. Wang et al. [9] observed that the degradation of IBU was directly affected by the pH of the solution, with the highest degradation rate (49.45%) in acidic pH values. The best photocatalytic yield for degradation at an acidic pH has been attributed to the adsorption of IBU on the surface of the catalysts due to the increase in electrostatic interactions between IBU and the photocatalyst [61].

#### 3.2.2. Effect of H_2_O_2_

**Figure 8:**
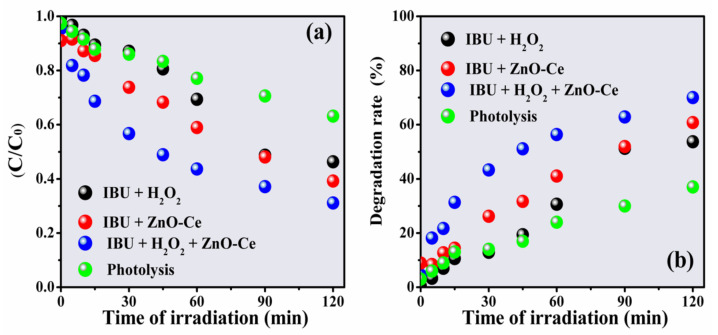
C/C_0_ rate for degradation of IBU in different systems and function of irradiation time (**a**) and degradation rate (**b**). Experimental conditions: IBU initial concentration: 20 ppm, photocatalyst: 0.50 gL^−1^, UV light: 125 W Hg without bulb, H_2_O_2_: 0.5 mmol/L^−1^.

Hydrogen peroxide is an oxidizing agent that, when reacting with photogenerated electrons on the semiconductor surface, forms hydroxyl radicals (^•^OH), and its use as an aid in the photodegradation process of pollutants is very promising [55]. However, conducting photodegradation with H_2_O_2_ produced an insufficient yield (52%) for total degradation (Figure 8). The degradation of IBU in this process is given by the reaction of hydroxyl radicals generated after the addition of hydrogen peroxide, as described by Equations (8)–(10) [9].

H_2_O_2_+ *h*v→ 2^•^OH (8)

H_2_O_2_ + O_2_^•−^→ OH^•^ + OH^−^ + O_2_ (9)

H_2_O_2_ + e^−^→ OH^•^ + OH^−^ (10)

The IBU degradation rate increased with the use of ZnO-Ce and ZnO-Ce in the presence of H_2_O_2_ systems (ZnO-Ce + H_2_O_2_) reaching about 60% and 70%, respectively. The improvement of the photochemical process can be linked to the interaction between the semiconductor and H_2_O_2_, increasing the photocatalyst sites and consequently facilitating the chemical interaction with IBU [61].

#### 3.2.3. Analyses of Reactive Oxidizing Species

**Figure 9:**
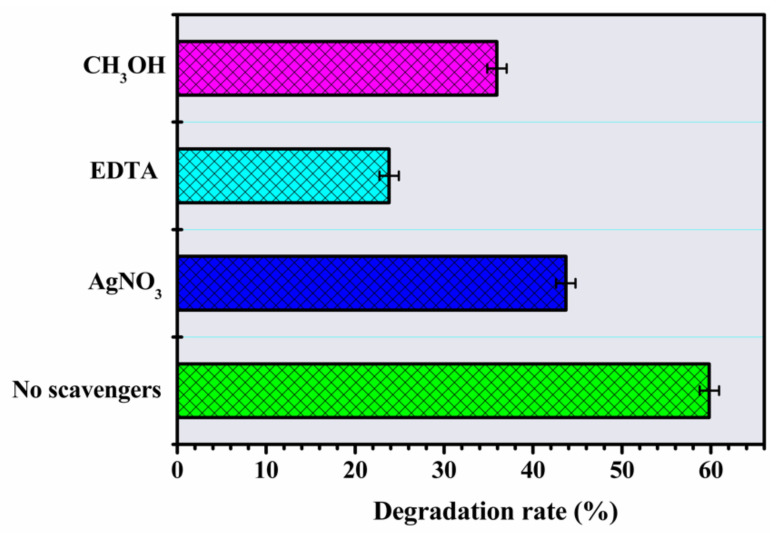
The effects of scavengers in degradation of IBU using ZnO-Ce (experimental conditions: IBU initial concentration: 20 ppm, photocatalyst: 0.50 gL^−1^, UV light: 125 W Hg without bulb, methyl alcohol: 3.4 × 10^−3^ mgL^−1^, silver nitrate: 5.0 × 10^−4^ mol/L and EDTA: 2.4 × 10^−6^ mol/L.

The degradation rate of IBU under the action of inhibitors was evaluated (Figure 9). In the presence of EDTA (h^+^, hole sequestrant), the decrease in degradation indicates the fundamental role of the participation of the holes, considering the substantialinhibition of the process (23%). The addition of AgNO_3_ (an electron scavenger) and CH_3_OH (an ^•^OH scavenger) reduced the degradation rate by 43% and 35% respectively. Therefore, the IBU degradation photoprocess seems to be more activated by the greater availability of h^+^ in VB, as well as other processes that help in the formation of the H_2_Oand ^•^OH radical [23,33]. These results contrast with the work of Xuet al. [54], who observed that the activity of the holes to generate oxidative species was less efficient in the IBU photodegradation mechanism. In the presence of Ag^+^ ions, the photocatalysis had the smallest decrease in total degradation, possibly due tothe deposition of Ag^0^ on the surface allowing better photocatalytic activity through the capture of photogenerated electrons avoiding recombination [32,62]. Overall, the order of photochemical activity in the photodegradation of IBU follows the order h^+^ > ^●^OH > e^−^.

#### 3.2.4. Reuse and Stability of the Photocatalyst

**Figure 10:**
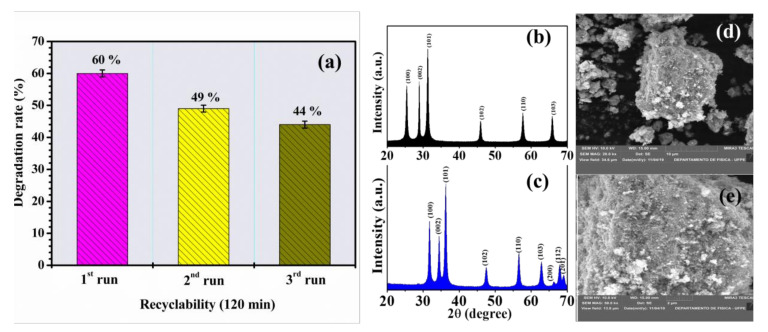
Reuse studies of IBU (20 ppm) using ZnO-Ce (0.50 gL^−1^) under UV light (**a**), ZnO XRD standards before irradiation (**b**), ZnO-Ce XRD standards after photocatalytic test (**c**), microscopy after the photocatalytic test of ZnO-Ce with 120 min under UV light (**d**,**e**).

The stability of a photocatalyst during application and after successive applications is extremely important [63,64]. Stability was assessed via consecutive reapplication of IBU degradation cycles. As shown in Figure 10a, after the third cycle, the compoundexhibited 44% degradation activity and maintained significant stability compared to the starting material (i.e., first degradation), reaching only 16% total loss, that is 11% (firstand secondrun) and 5% (second and thirdrun), which allows considering good kinetic stability. The equivalent loss value demonstrates that the combination of ZnO-Ce has a significantly active photocatalytic performance; however, any structural and morphological changes are important to observe.

To verify the stability of the photocatalyst, it was examined before and after the photocatalytic reaction through the evaluation of crystallinity (XRD) and microscopy (SEM) (Figure 10b–e). After the photocatalytic cycle, ZnO-Ce exhibited characteristic diffraction peaks, indicating that the precursor photocatalyst maintained its parent crystal structure with small displacements relative to ZnO and similar toZnO-Ce before testing (observation discussed in Section 3.1).

Microscopic images were also recorded to assess morphological stability (Figure 10c,d). The typical morphology of ZnO-Ce with irregular shapes and agglomerates prevailed even after the photocatalytic test, ensuring stability viathe cerium that maintains the disorganization of the spherical structure observed only in ZnO. 

XRD and SEM studies confirmed that the interaction between IBU and ZnO-Ce (under UV light) resulted in a strong interaction that guaranteed photocatalytic activity; thus, they are considered promising and stable for future applications in the photodegradation of different classes of pollutants. A previous study reinforces that recyclability tests have been used in several studies to direct such photocatalysts towardsenvironmental applications [64].

Previousauthors have evaluated material stability, such as Araújo et al., who investigated material stability through reuse/recycles in photocatalytic tests using Zno synthesized in the presence of polysaccharides [33]. Sa et al. [24] evaluated this parameter inthe degradation of a bentazone herbicide. In order to estimate the photocatalytic activity of ZnO and Ce nanorods under the same conditions with the best 1% CZ sample, Kardes et al. [65] observed three consecutive runs of color removal in AR88 azo dye. They observed a slight decrease in the percentage performance in each run, and the reason for the decrease was investigated by the ratio of ZnO leaching in the dye solution.

Saad et al. [66] investigated CH/ZnO and CH/Ce–ZnO nano-flowers that showed high stability and could be effectively reused through five consecutive cycles. For the reuse tests, CH/ZnO values of 100%, 100%, 95.4%, 90.3%, and 84% were obtained for cycle 1, cycle 2, cycle 3, cycle 4, and cycle 5, respectively. For CH/Ce-ZnO, the degradation percentages for cycle 1, cycle 2, cycle 3, cycle 4, and cycle 5 were100%, 100%, 97.2%, 92%, and 90%, respectively. All percentages reflected excellent stability and concluded that the doping of ZnO with Ce contributed to the stability of the composites.

### 3.3. Toxicological Monitoring of Aqueous Solutions by Artemia salina

**Figure 11:**
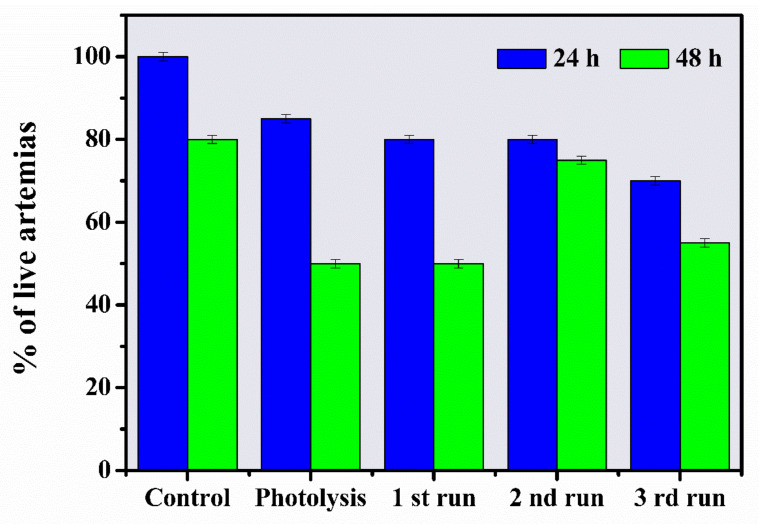
Bioassay using *A. Salina*, with control corresponding to microcrustaceans cultivated in synthetic saline solution (38 gL^−1^), followed by photolytic, photocatalytic (firstrun), and after successive cycles (secondand thirdruns).

*Artemia salina*is abioindicator widely used for the toxicological evaluation of several pollutants [67]. Toxicity was evaluated in solutions before and after the photocatalytic treatment to observe the toxicological effect of ZnO-Ce as an environmental photocatalyst, that is, one that promotes both the degradation of the drug and the reduction of toxicity. Figure 11 presents the percentages found in the toxicity evaluation through the percentage of live *Artemia*, which guarantees the survival of the nauplii in the solutions. First, the control trial demonstrated 100% survival of *Artemias* at 24 h and 80% after 48 h. A reduced percentage of mortality was observed for all irradiated solutions, such as the photolytic solution (15%—24 h; 50%—8 h), the photocatalytic solution or first run (20%—24 h; 50%—48 h), and the reused solutions (20%—24 h; 25% at 48 h—second run) and (30%—24 h; 45%—48 h—third run). This behavior may be due to the derivation of photo-generated by-products during the irradiation process, which meantthe solution was able to cause mortality. However, the formation of these by-products of an unknown nature did not significantly compromise the survival of the bioindicator, since the solution only presented 45% toxicity and/or mortality 48 h after the third cycle, which ensured standardization within the lethal dose (LD 50), even if only the photogenerated solution is evaluated. Given the 50% survival in the last stage tested, it is probablethat no more toxic metabolites were generated in relation to the starting drug. According to Reddy et al. [68] and Zakari et al. [69], effluents alter their physicochemical propertieswhen released into aquatic matrices, allowing the effluent to be more or less miscible depending on its concentration in the water. Therefore, ZnO-Ce has photocatalyst potential for the decontamination of drug-containing effluents and did not show toxicity inthe photogenerated solutions. 

## 4. Conclusions

X-ray diffraction showed that the photocatalyst is crystalline and has a hexagonal wurtzite structure, and micrographs illustrated that the morphology of ZnO-Ceis spherical in shape. ZnO-Ce exhibited high photocatalytic performance towards IBU degradation under UV light. The best experimental condition for IBU photodegradation was obtained in an acidic pH using 0.50 gL^−1^ of ZnO-Ce in a solution of 20 ppm of IBU. The effect of pH can be attributed to the protonation/deprotonation of the catalyst surface, altering the generation and activity of the hydroxyl radical and, consequently, decreasing the degradation rate. The presence of hydrogen peroxide favored the photocatalysis process. In addition, the degradation efficiency of IBU was decreased in the presence of EDTA, indicating that the hole plays a key role in the degradation process of ibuprofen. The IBU degradation rate decreased by 16% after three photochemical cycles, and XRD analysis indicated structural stability of ZnO-Ce at the end of the photoreactions. The non-toxicity of the irradiated solutions evaluated against *Artemia salina* produced a low percentage of mortality. A deeper understanding of the photogenerated by-products and the proposal ofan appropriate mechanism based on the identification of intermediates is an important step that needs to be investigated in future work, as well as the antimicrobial activity of this photocatalytic material. In this study, anindication of the formation of fewertoxic by-products was shown, as presented in the toxicity test, indicating an interesting system for future applications in the treatment of wastewater.

## Figures and Tables

**Table 1:** Specific surface area of ZnO-Ce and otherphotocatalysts.

Material	Specific Surf. Area (m^2^/g)	Referential
ZnO:Ce^4+^	24.807	Experimental
0.3% Ce/ZnO	93.8	[48]
CZO-1	39.37	[50]
CZ6	30.6	[49]
3% Ce—3% Y-ZnO	104.5	[51]
0.1 Ce/ZnO	3.1	[47]
0.25 Ce/ZnO	3.8	
0.5 Ce/ZnO	6.5	
3 Ce/ZnO	11.6	
5 Ce/ZnO	16.1	
1% Ce/ZnO	4.189	[52]
3% Ce/ZnO	10.384	
5% Ce/ZnO	12.899	
8% Ce/ZnO	17.303	

## Data Availability

Data sharing is not applicable to this article.

## Supplementary Materials

The following are available online at https://www.mdpi.com/article/10.3390/ma14195891/s1, Figure S1: The mapping EDS and elemental mapping of ZnO-Ce.

## References

[1] Bade R., White J.M., Gerber C. (2017). Qualitative and quantitative temporal analysis of licit and illicit drugs in wastewater in Australia using liquid chromatography coupled to mass spectrometry. Anal. Bioanal. Chem..

[2] Im J.K., Hwang M.Y., Lee E.H., Noh H.R., Yu S.J. (2020). Pharmaceutical compounds in tributaries of the Han River watershed, South Korea. Environ. Res..

[3] Chopra S., Kumar D. (2020). Ibuprofen as an emerging organic contaminant in environment, distribution and remediation. Heliyon.

[4] Rastogi T., Mahmoud W., Kümmerer K. (2018). Human and veterinary drugs in the environment. Encyclopedia of the Anthropocene.

[5] Rosenfeld P.E., Feng L.G.H. (2011). Risks of Hazardous Wastes.

[6] Couto C.F., Lange L., Amaral M.C. (2019). Occurrence, fate and removal of pharmaceutically active compounds (PhACs) in water and wastewater treatment plants—A review. J. Water Process. Eng..

[7] Jiménez-Salcedo M., Monge M., Tena M.T. (2019). Photocatalytic degradation of ibuprofen in water using TiO_2_/UV and g-C3N4/visible light: Study of intermediate degradation products by liquid chromatography coupled to high-resolution mass spectrometry. Chemosphere.

[8] Jallouli N., Pastrana-Martínez L.M., Ribeiro A.R., Moreira N.F.F. (2018). Heterogeneous photocatalytic degradation of ibuprofen in ultrapure water, municipal and pharmaceutical industry wastewaters using a TiO_2_/UV—LED system. Chem. Eng. J..

[9] Wang Z., Srivastava V., Ambat I., Safaei Z., Sillanpää M. (2019). Degradation of ibuprofen by UV-LED/catalytic advanced oxidation process. J. Water Process Eng..

[10] Carballa M., Omil F., Lema J.M., Llompart M., García-Jares C., Rodríguez I., Gómez M., Ternes T. (2004). Behavior of pharmaceuticals, cosmetics and hormones in a sewage treatment plant. Water Res..

[11] Honorio L., Trigueiro P., Viana B.C., Ribeiro A.B., Osajima J.A. (2019). Nanostructured materials for the photocatalytic degradation of organic pollutants in water. Nanostructured Materials for Treating Aquatic Pollution.

[12] Huang Y., Liang M., Ma L., Wang Y., Zhang D., Li L. (2021). Ozonation catalysed by ferrosilicon for the degradation of ibuprofen in water. Environ. Pollut..

[13] Sun S., Yao H., Fu W., Liu F., Wang X., Zhang W. (2021). Enhanced degradation of carbamazepine in FeOCl based photo-fenton reaction. J. Environ. Chem. Eng..

[14] Núñez-Flores A., Sandoval A., Mancilla E., Hidalgo-Millán A., Ascanio G. (2020). Enhancement of photocatalytic degradation of ibuprofen contained in water using a static mixer. Chem. Eng. Res. Des..

[15] Araujo F.P., Tadeu I., Batista S., Almeida L.R.D., Brito G.D.C., Dittz D., Cavalcanti E., Filho S., Osajima J.A., Lobo O. (2020). Printing composite nano fi laments for use in a simple andlow-cost 3D pen. J. Mater. Res..

[16] Hanh N.T., Tri N.L.M., Van Thuan D., Tung M.H.T., Pham T.-D., Minh T.D., Trang H.T., Binh M.T., Nguyen M.V. (2019). Monocrotophos pesticide effectively removed by novel visible light driven Cu doped ZnO photocatalyst. J. Photochem. Photobiol. A Chem..

[17] Ahmad I., Akhtar M.S., Ahmed E., Ahmad M., Keller V., Khan W.Q., Khalid N. (2020). Rare earth co-doped ZnO photocatalysts: Solution combustion synthesis and environmental applications. Sep. Purif. Technol..

[18] Abebe B., Murthy H.A., Amare E. (2020). Enhancing the photocatalytic efficiency of ZnO: Defects, heterojunction, and optimization. Environ. Nanotechnol. Monit. Manag..

[19] Shah A.A., Bhatti M.A., Tahira A., Chandio A., Channa I.A., Sahito A.G., Chalangar E., Willander M., Nur O., Ibupoto Z.H. (2020). Facile synthesis of copper doped ZnO nanorods for the efficient photo degradation of methylene blue and methyl orange. Ceram. Int..

[20] Shirdel B., Behnajady M.A. (2020). Visible-light-induced degradation of Rhodamine B by Ba doped ZnO nanoparticles. J. Mol. Liq..

[21] Al Abri R., Al Marzouqi F., Kuvarega A.T., Meetani M., Al Kindy S.M., Karthikeyan S., Kim Y., Selvaraj R. (2019). Nanostructured cerium-dopedZnO for photocatalytic degradation of pharmaceuticals in aqueous solution. J. Photochem. Photobiol. A Chem..

[22] Babu D.S., Srivastava V., Nidheesh P., Kumar M.S. (2019). Detoxification of water and wastewater by advanced oxidation processes. Sci. Total Environ..

[23] Araujo F.P., Trigueiro P., Honório L.M.C., Furtini M.B., Oliveira D.M., Almeida L.C., Garcia R.R.P., Viana B.C., Silva-Filho E.C., Osajima J.A. (2020). A novel green approach based on ZnO nanoparticles and polysaccharides for photocatalytic performance. Dalton Trans..

[24] Osajima J.A., Sá A.S., Honorio L.M.C., Trigueiro P., Pinto L.I.F., Oliveira J.A., Furtini M.B., Bezerra R.D.S., Alcantara A.C.S., Silva-Filho E.C. (2021). Au@Ag bimetallic nanoparticles deposited on palygorskite in the presence of TiO_2_ for enhanced photodegradation activity through synergistic effect. Environ. Sci. Pollut. Res..

[25] Cai H., Liang J., Ning X.-A., Lai X., Li Y. (2020). Algal toxicity induced by effluents from textile-dyeing wastewater treatment plants. J. Environ. Sci..

[26] Nunes B.S., Carvalho F., Guilhermino L., Van Stappen G. (2006). Use of the genus *Artemia* in ecotoxicity testing. Environ. Pollut..

[27] Araujo F.P., Honorio L., Lima I.S., Trigueiro P., Almeida L., Fechine P., dos Santos F.E.P., Peña-Garcia R., Silva-Filho E.C., Osajima J.A. (2020). New composite TiO_2_/naturals gums for high efficiency in photodiscoloration process. Ceram. Int..

[28] Bekhit F., Farag S., Attia A.M. (2020). Decolorization and degradation of the Azo dye by bacterial cells coated with magnetic iron oxide nanoparticles. Environ. Nanotechnol. Monit. Manag..

[29] Dias J.D.S., Batista F.R.M., Bacani R., Triboni E.R. (2020). Structural characterization of SnO nanoparticles synthesized by the hydrothermal and microwave routes. Sci. Rep..

[30] Makuła P., Pacia M., Macyk W. (2018). How to correctly determine the band gap energy of modified semiconductor photocatalysts based on UV–vis spectra. J. Phys. Chem. Lett..

[31] Christy E.J.S., Amalraj A., Rajeswari A., Pius A. (2021). Enhanced photocatalytic performance of Zr(IV) doped ZnO nanocomposite for the degradation efficiency of different azo dyes. Environ. Chem. Ecotoxicol..

[32] Teixeira A.R.F.A., Neris A., Longo E., Filho J.R.D.C., Hakki A., Macphee D., dos Santos I.M.G. (2019). SrSnO_3_ perovskite obtained by the modified Pechini method—insights about its photocatalytic activity. J. Photochem. Photobiol. A Chem..

[33] Araujo F.P., Trigueiro P., Honório L.M.C., Oliveira D.M., Almeida L.C., Garcia R.P., Lobo A.O., Cantanhêde W., Silva-Filho E.C., Osajima J.A. (2020). Eco-friendly synthesis and photocatalytic application of flowers-like ZnO structures using Arabic and Karaya Gums. Int. J. Biol. Macromol..

[34] Meyer B.N., Ferrigni N.R., Putnam J.E., Jacobsen L.B., Nichols D.E., McLaughlin J.L. (1982). Brine shrimp: A convenient general bioassay for active plant constituents. Planta Med..

[35] Babar U., Garad N., Mohite A., Babar B., Shelke H., Kamble P., Pawar U. (2021). Study the photovoltaic performance of pure and Cd-doped ZnO nanoparticles prepared by reflux method. Mater. Today Proc..

[36] Sangeeta M., Karthik K., Ravishankar R., Anantharaju K., Nagabhushana H., Jeetendra K., Vidya Y., Renuka L. (2017). Synthesis of ZnO, MgO and ZnO/MgO by solution combustion method: Characterization and photocatalytic studies. Mater. Today Proc..

[37] Castro-Lopes S., Guerra Y., Silva-Sousa A., Oliveira D., Gonçalves L., Franco A., Padrón-Hernández E., Peña-Garcia R. (2020). Influence of pH on the structural and magnetic properties of Fe-doped ZnO nanoparticles synthesized by sol gel method. Solid State Sci..

[38] Shannon R.D. (1976). Revised effective ionic radii and systematic studies of interatomic distances in halides and chalcogenides. Acta Crystallogr. A.

[39] Neupane G.R., Kaphle A., Hari P. (2019). Microwave-assisted Fe-doped ZnO nanoparticles for enhancement of silicon solar cell efficiency. Sol. Energy Mater. Sol. Cells.

[40] Fifere N., Airinei A., Timpu D., Rotaru A., Sacarescu L., Ursu L. (2018). New insights into structural and magnetic properties of Ce doped ZnO nanoparticles. J. Alloys Compd..

[41] Caregnato P., Jiménez K.R.E., Villabrille P.I. (2021). Ce-doped ZnO as photocatalyst for carbamazepine degradation. Catal. Today.

[42] Bechambi O., Touati A., Sayadi S., Najjar W. (2015). Effect of cerium doping on the textural, structural and optical properties of zinc oxide: Role of cerium and hydrogen peroxide to enhance the photocatalytic degradation of endocrine disrupting compounds. Mater. Sci. Semicond. Process..

[43] Khanlary M.R., Hajinorozi A., Baghshahi S. (2015). Influence of Ce doping concentration on the structural and optical properties of sol-gel derived ZnO:Ce nanostructures. J. Inorg. Organomet. Polym. Mater..

[44] Janotti A., Van de Walle C.G. (2009). Fundamentals of zinc oxide as a semiconductor. Rep. Prog. Phys..

[45] Yousefi M., Amiri M., Azimirad R., Moshfegh A. (2011). Enhanced photoelectrochemical activity of Ce doped ZnO nanocomposite thin films under visible light. J. Electroanal. Chem..

[46] Zhang L., Lv F., Zhang W., Li R., Zhong H., Zhao Y., Zhang Y., Wang X. (2009). Photo degradation of methyl orange by attapulgite-SnO_2_-TiO_2_ nanocomposites. J. Hazard. Mater..

[47] Faisal M., Ismail A.A., Ibrahim A., Bouzid H., Al-Sayari S.A. (2013). Highly efficient photocatalyst based on Ce doped ZnO nanorods: Controllable synthesis and enhanced photocatalytic activity. Chem. Eng. J..

[48] Jiang J., Zhang K., Chen X., Zhao F., Xie T., Wang D., Lin Y. (2017). Porous Ce-doped ZnO hollow sphere with enhanced photodegradation activity for artificial waste water. J. Alloys Compd..

[49] Chand P., Singh V., Kumar D. (2020). Rapid visible light-driven photocatalytic degradation using Ce-doped ZnOnanocatalysts. Vacuum.

[50] Zhang Y.-H., Peng M.-X., Yue L.-J., Chen J.-L., Gong F.-L., Xie K.-F., Fang S.-M. (2021). A room-temperature aniline sensor based on Ce doped ZnO porous nanosheets with abundant oxygen vacancies. J. Alloys Compd..

[51] Ahmad I., Akhtar M.S., Manzoor M.F., Wajid M., Noman M., Ahmed E., Ahmad M., Khan W.Q., Rana A.M. (2021). Synthesis of yttrium and cerium doped ZnO nanoparticles as highly inexpensive and stable photocatalysts for hydrogen evolution. J. Rare Earths.

[52] Wang L., Ji Z., Lin J., Li P. (2017). Preparation and optical and photocatalytic properties of Ce-doped ZnO microstructures by simple solution method. Mater. Sci. Semicond. Process..

[53] Mafa P.J., Swana U.S., Liu D., Gui J., Mamba B.B., Kuvarega A.T. (2021). Synthesis of Bi_5_O_7_I-MoO_3_ photocatalyst via. simultaneous calcination of BiOI and MoS_2_ for visible light degradation of ibuprofen. Colloids Surf. A Physicochem. Eng. Asp..

[54] Xu P., Zheng D., Xie Z., He Q., Yu J. (2020). The degradation of ibuprofen in a novel microbial fuel cell with PANi@CNTs/SS bio-anode and CuInS2 photocatalytic cathode: Property, efficiency and mechanism. J. Clean. Prod..

[55] Akpan U., Hameed B.H. (2009). Parameters affecting the photocatalytic degradation of dyes using TiO_2_-based photocatalysts: A review. J. Hazard. Mater..

[56] De Oliveira W.V., Morais A., Ícaro S., Honorio L., Trigueiro P., Almeida L., Garcia R.R.P., Viana B., Furtini M.B., Silva-Filho E.C. (2020). TiO_2_ immobilized on fibrous clay as strategies to photocatalytic activity. Mater. Res..

[57] Islam M.R., Rahman M., Farhad S.F.U., Podder J. (2019). Structural, optical and photocatalysis properties of sol-gel deposited Al-doped ZnO thin films. Surf. Interfaces.

[58] Bomila R., Srinivasan S., Venkatesan A., Bharath B., Perinbam K. (2017). Structural, optical and antibacterial activity studies of Ce-doped ZnO nanoparticles prepared by wet-chemical method. Mater. Res. Innov..

[59] Anwer H., Mahmood A., Lee J., Kim K.-H., Park J.-W., Yip A. (2019). Photocatalysts for degradation of dyes in industrial effluents: Opportunities and challenges. Nano Res..

[60] Ren Z., Romar H., Varila T., Xu X., Wang Z., Sillanpää M., Leiviskä T. (2021). Ibuprofen degradation using a Co-doped carbon matrix derived from peat as a peroxymonosulphate activator. Environ. Res..

[61] Monteoliva-García A., Martín-Pascual J., Muñío M.M., Poyatos J.M. (2019). Removal of carbamazepine, ciprofloxacin and ibuprofen in real urban wastewater by using light-driven advanced oxidation processes. Int. J. Environ. Sci. Technol..

[62] Honorio L.M.C., de Oliveira A.L.M., Filho E.C.D.S., Osajima J.A., Hakki A., Macphee D.E., dos Santos I.M.G. (2020). Supporting the photocatalysts on ZrO_2_: An effective way to enhance the photocatalytic activity of SrSnO_3_. Appl. Surf. Sci..

[63] Ahmad I., Shukrullah S., Ahmad M., Ahmed E., Naz M.Y., Akhtar M.S., Khalid N., Hussain A., Hussain I. (2021). Effect of Al doping on the photocatalytic activity of ZnO nanoparticles decorated on CNTs and graphene: Solvothermal synthesis and study of experimental parameters. Mater. Sci. Semicond. Process..

[64] Si Y., Chen Y., Fu Y., Zhang X., Zuo F., Zhang T., Yan Q. (2020). Hierarchical self-assembly of graphene-bridged on AgIO_3_/BiVO_4_: An efficient heterogeneous photocatalyst with enhanced photodegradation of organic pollutant under visible light. J. Alloys Compd..

[65] Kardeş M., Dindaş G.B., Yatmaz H.C., Dizge N., Öztürk K. (2020). CBD grown pure and Ce-doped ZnO nanorods: Comparison of their photocatalytic degrading efficiencies on AR88 azo dye under visible light irradiation. Colloids Surf. A Physicochem. Eng. Asp..

[66] Saad A.M., Abukhadra M.R., Ahmed S.A.-K., Elzanaty A.M., Mady A.H., Betiha M., Shim J.-J., Rabie A.M. (2020). Photocatalytic degradation of malachite green dye using chitosan supported ZnO and Ce-ZnO nano-flowers under visible light. J. Environ. Manag..

[67] De Andrade F., de Lima G., Augusti R., da Silva J., Coelho M., Paniago R., Machado I. (2015). A novel TiO_2_/autoclaved cellular concrete composite: From a precast building material to a new floating photocatalyst for degradation of organic water contaminants. J. Water Process. Eng..

[68] Reddy S., Osborne W.J. (2020). Heavy metal determination and aquatic toxicity evaluation of textile dyes and effluents using *Artemia salina*. Biocatal. Agric. Biotechnol..

[69] Zakari A., Kubmarawa D. (2016). Acute toxicity (LD50) studies using swiss albino mice and brine shrimp lethality (LC50 and LC90) determination of the ethanol extract of stem bark of *Echinaceaeangustifolia* DC. Nat. Prod. Chem. Res..

