# Supplementary material for: A Brief Photocatalytic Study of ZnO Containing Cerium towards Ibuprofen Degradation
Source: Materials (Basel). 2021 Oct 8;14(19):5891. doi: 10.3390/ma14195891 (PMC8510120; doi:10.3390/ma14195891)
Supplement: Supplementary file 1 [file materials-14-05891-s001.zip › materials-1370882-supplementary.pdf]

materials

Supplementary Materials

A Brief Photocatalytic Study of ZnO Containing Cerium Towards Ibuprofen Degradation

Alexandro S. Sá [1], Rodrigo P. Feitosa [1], Luzia Honório [1], Ramón Peña-Garcia [1,2], Luciano C. Almeida [3], Juliana S. Dias [4], Lorena P. Brazuna [4], Thiago G. Tabuti [4], Eduardo R. Triboni [4], Josy A. Osajima [1,*] and Edson C. da Silva-Filho [1,*]

[1] LIMAV, Interdisciplinary Laboratory for Advanced Materials, Ministro Petronio Portela, Federal University of Píaui, Teresina, Piaui 64049-550, Brazil; alexsousasa@hotmail.com (A.S.S.); rooprado@ufpi.edu.br (R.P.F.);luzia_quimica@yahoo.com.br (L.H.);rraudelp@gmail.com(R.P.-G.)
[2] Federal Rural University of Pernambuco, Academic Unit of Santo Agostinho, Recife, Pernambuco, 52171-900 Brazil; luciano.calmeida@ufpe.br
[3] Chemical Engineering Department,Federal University of Pernambuco, Recife, Pernambuco, 52171-900, Brazil
[4] Laboratory of Nanotechnology and Process Engineering, Chemistry Engineering Department, University of São Paulo, Lorena 12602-810, Brazil; julianasilvaddias@gmail.com (J.S.D.); lorenaamelo1@gmail.com (L.P.B.); thiagogaleote@gmail.com (T.G.T.); tribonier@usp.br (E.R.T.)
* Correspondence:josyosajima@ufpi.edu.br (J.A.O.);edsonfilho@ufpi.edu.br (E.C.d.S.-F.)

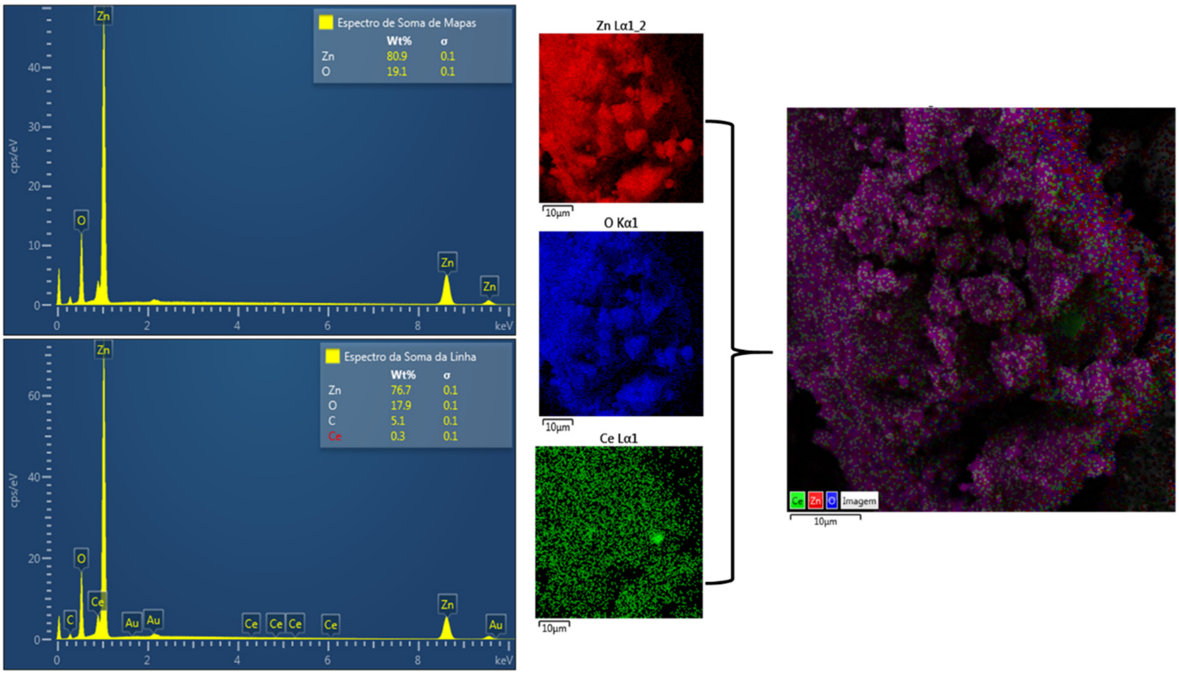

Figure S1. The mapping EDS and elemental mapping of ZnO-Ce.
